# Effectiveness of sucroferric oxyhydroxide in patients on *on-line* hemodiafiltration in real-world clinical practice: A retrospective study

**DOI:** 10.1590/2175-8239-JBN-2018-0142

**Published:** 2019-02-04

**Authors:** Aníbal Ferreira, Bruno Pinto, David Navarro, João Aniceto, Pedro L Neves, Pedro Ponce

**Affiliations:** 1 Universidade Nova de Lisboa Nova Medical School Lisboa Portugal Universidade Nova de Lisboa, Nova Medical School, Lisboa, Portugal.; 2 NephroCare Portugal Fresenius Medical Care Portugal Lisboa Portugal NephroCare Portugal, Fresenius Medical Care Portugal, Lisboa, Portugal.; 3 NephroCare Vila Franca de Xira Vila Franca de Xira Portugal NephroCare Vila Franca de Xira, Vila Franca de Xira, Portugal.; 4 NephroCare Évora Évora Portugal NephroCare Évora, Évora, Portugal.; 5 Centro Hospitalar Universitário do Algarve Faro Portugal Centro Hospitalar Universitário do Algarve, Faro, Portugal; 6 NephroCare Lumiar Lisboa Portugal NephroCare Lumiar, Lisboa, Portugal.; 7 NephroCare Portugal Lisboa Portugal NephroCare Portugal, Lisboa, Portugal.

**Keywords:** Sucroferric Oxyhydroxide, Hyperphosphatemia, Phosphorus, Renal Insufficiency, Chronic, Hemodiafiltration

## Abstract

**Introduction:**

Hyperphosphatemia is a serious consequence of chronic kidney disease and has been associated with an increased risk for cardiovascular disease. Controlling serum phosphorus levels in patients on dialysis is a challenge for the clinicians and implies, in most cases, the use of phosphate binders (PB). Part of the reason for this challenge is poor adherence to treatment because of the high pill burden in this patient group.

**Objective:**

To assess the real-world effectiveness of sucroferric oxyhydroxide (SO) in controlling serum phosphorus levels and determine the associated pill burden.

**Methods:**

A multicenter, quantitative, retrospective, before-after study was conducted with patients receiving online hemodiafiltration. Patients who switched to SO as a part of routine care were included in the study. PB treatment, number of pills, serum phosphorus levels, and intravenous iron medication and dosage were collected monthly during the six months of treatment with either PB or SO.

**Results:**

A total of 42 patients were included in the study. After switching from a PB to SO, the prescribed pills/day was reduced 67% from 6 pills/day to 2 pills/day (*p* < 0.001) and the frequency of pill intake was lowered from 3 times/day to 2 times/day (*p* < 0.001). During the treatment with SO, the proportion of patients with serum phosphorus ≤ 5.5 mg/dL increased from 33.3% at baseline to 45% after six months of treatment.

**Conclusion:**

During the six-month follow-up with SO, serum phosphorus levels were controlled with one third of the pills/day compared to other PB.

## Introduction

Hyperphosphatemia is a serious and common consequence of chronic kidney disease (CKD) and has been associated with an increased risk for cardiovascular disease.^1^^,^^2^ According to the Kidney Disease: Improving Global Outcomes (KDIGO) guidelines, the serum phosphorus levels should be as close as possible to normal values to improve the clinical outcome. Restrictions for dietary phosphate intake and dialysis are frequently insufficient to remove excess serum phosphorus and prevent hyperphosphatemia. Hence, patients with CKD undergoing chronic dialysis frequently require treatment with phosphate binders (PB), such as sevelamer carbonate, calcium acetate, calcium acetate/magnesium carbonate, or lanthanum carbonate, to achieve recommended serum phosphorus levels.^3^ In addition, treatment with PB has been associated with a reduction in the all-cause mortality rate.^4^^-^6 However, patients on dialysis generally experience a high pill burden, approximately half of which is due to PB.^7^ A high number of prescribed pills is associated with lower adherence to medication and worse serum phosphorus control.^8^

Sucroferric oxyhydroxide (SO) is a new, iron-based compound with a high phosphate binding capacity.^9^ The efficacy of SO has been demonstrated in clinical trials in patients with CKD.^10^^-^12 In phase III studies, SO was shown to reduce serum phosphorus levels to the same extent as other PB such as sevelamer carbonate but with a substantial reduction of pill burden.^10^^,^^11^ In addition, the iron uptake in the gastrointestinal tract has been described as minimal^9^ and SO is well tolerated.^9^^,^^10^^,^^13^

SO was recently introduced in Portugal and, to our knowledge, this is the first study to investigate its beneficial effects in clinical practice. Therefore, in the present study, we assessed the real-world effectiveness of SO on reducing the serum phosphorus levels and the number of pills per day during the first six months of treatment.

## Methods

This retrospective before-after study was based on patients receiving online hemodiafiltration, treated at three dialysis units located at three geographic areas in Portugal (NephroCare units in Évora, Faro, and Vila Franca de Xira). Deidentified data were extracted from patient medical records. Eligible patients were at least 18 years old, had previously been prescribed a PB different from SO for at least six months, and due to maintenance of uncontrolled high serum phosphorus levels, were changed to SO therapy, when this medication was made available in Portugal. All patients from the three dialysis units that completed six months of SO therapy were included in the present evaluation. Data were collected during November 2016 for patients who received their first treatment with SO between January 2015 and April 2016. The sample size was limited by the number of patients meeting the inclusion criteria. Medication prescription and dosage were made by attending nephrologists as a part of their routine care. The treatment periods were defined as an initial six-month period of treatment with a PB (sevelamer carbonate, calcium acetate, or calcium acetate/magnesium carbonate), followed by a washout phase of one month, after which the patients received treatment with SO for an additional six-month period. Baseline was defined as the first month of treatment with PB or SO, respectively. Data on demographic characteristics (age and sex), dry weight, and dialysis time were assessed at the beginning of the first month of treatment with PB. Age-adjusted Charlson comorbidity index was calculated at the beginning of the first month of treatment with PB. Previous PB treatment, number of pills, and intravenous (IV) iron medication and dosage were collected monthly during the six months of treatment with either PB or SO. Serum levels of ferritin, hemoglobin, calcium, phosphorus, and intact parathyroid hormone (iPTH) were collected at baseline and every month up to six months of treatment with either PB or SO. Clinical parameters were measured using standardized laboratory tests.

All patients were evaluated monthly using body bio-impedance to define body composition compartments, hydration, and muscular composition. In addition, a monthly evaluation by the clinics’ dietitian was performed for each patient. Serum albumin levels were determined every three months. No significant variation was observed in dietary habits, nutrition status, and bio-impedance results during the 12 months period.

Categorical variables were presented as relative and absolute frequencies and compared using chi-squared test or Fisher´s Exact test. Continuous variables were presented as mean and standard deviation, or median and range, and compared using chi-squared test or Fisher´s exact test. Shapiro-Wilk test was used to assess the normality of continuous variables. To assess differences between PB and SO in hemoglobin, ferritin, calcium, iPTH, and phosphorus serum levels, a General Linear Mixed Model (GLMM) with treatment as fixed factor and subject and timepoint as random effects was fitted to the data. Maximum likelihood using the Nelder-Mead optimizer was used for parameter estimation. McNemar’s test was used for the comparison of baseline between proportion of subjects with phosphorus ≤ 5.5 mg/dL. All statistical analyses were conducted using R Statistical Software version 3.4.1.

This study was reviewed and approved by Institutional Review Board (NephroCare Portugal). The study was conducted in accordance with the Declaration of Helsinki.

## Results

A total of 42 patients distributed over three centers were included in the study. The mean age was 53.2 years and the median time on dialysis was 76.5 months. Patients were previously prescribed calcium acetate/magnesium carbonate (42.9%), sevelamer carbonate (31.0%), or calcium acetate (19.0%). For three of the patients (7.1%), data on the type of PB initially prescribed were missing. Patient demographic and baseline characteristics are shown in Table 1.

**Table 1:** Baseline characteristics for patient cohort

Characteristics	Patients (N = 42)
Gender, n (%)	
Male	28 (66.7)
Age, mean (SD), years	53.2 (13.2)
≥ 50 years, n (%)	26 (61.9)
Dry weight, mean (SD), kg	70.4 (13.9)
≥ 70 kg, n (%)	22 (52.4)
Phosphate binder, n (%)	
Calcium acetate/Magnesium carbonate	18 (42.9)
Sevelamer carbonate	13 (31.0)
Calcium acetate	8 (19.0)
Unknown/Not Reported	3 (7.1)
Dialysis time, median (minimum-maximum), months	76.5 (18.0 - 406.0)
Age adjusted Charlson Index, median (minimum-maximum)	4.0 (2.0 - 11.0)

Clinical parameters measured at baseline, and at three- and six-months post-treatment with PB or SO are summarized in Table 2. Patients with a recorded PB were prescribed a median of 6 pills/day. After switching to SO, the pill burden was reduced by 67% to 2 pills/day (Table 2). The frequency of pill intake was lowered from 3 times/day to 2 times/day after switching to SO (Table 2). The mean IV iron dose decreased (from 2.9 to 2.4 mg/dL per month) and a reduction in the number of patients on iron therapy (78.6% versus 59.5%) was observed after switching to SO; however, the changes were not statistically significant (Table 2). The serum levels of ferritin, hemoglobin, calcium, and iPTH was measured at baseline, and at three- and six-months post-treatment with PB or SO (Table 2). However, when comparing PB and SO at any of the timepoints there was no statistically significant differenc for any of the clinical parameters (Table 2). Serum phosphorus levels were also measured at baseline, and at three- and six-months post-treatment with PB or SO. Serum phosphorus levels changed from 5.8 mg/dL at baseline to 6.0 mg/dL after six months of treatment with a PB. During the follow-up with SO, the serum phosphorus levels decreased from 6.0 mg/dL at baseline to 5.7 mg/dL after six months of treatment (Table 2). However, the difference between the two treatments at any of the timepoints was not statistically significant (Table 2).

**Table 2:** Comparison of changes in pill intake and clinical parameters at baseline and at three- and six- months post-treatment.

Parameter	Phosphate Binders		Sucroferric Oxyhydroxide		*p* value
	n (%)	Mean (SD)	n (%)	Mean (SD)	
Pill Burden					
Prescribed pills/day	39 (92.9)	6 (2 - 15)*	42 (100)	2 (1 - 6)*	< 0.001
Frequency	39 (92.9)	3 (1 - 4)*	42 (100)	2 (1 - 3)*	< 0.001
Ferritin, ng/mL					0.567
Baseline	16 (38.1)	359.5 (177.2)	17 (40.5)	426.0 (253.3)	0.601
3 MPT	24 (57.1)	511.4 (279.4)	14 (33.3)	421.2 (159.9)	0.205
6 MPT	23 (54.8)	479.7 (269.8)	12 (28.6)	491.8 (233.9)	0.384
IV Iron, g^†^					-
6 MPT	33 (78.6)	2.9 (3.1)	25 (59.5)	2.4 (2.7)	0.342
Hemoglobin, g/dL					0.247
Baseline	42 (100)	11.1 (1.3)	42 (100)	11.2 (1.6)	0.886
3 MPT	42 (100)	11.1 (1.2)	42 (100)	11.2 (1.6)	0.666
6 MPT	41 (97.6)	11.2 (1.5)	42 (100)	11.2 (1.6)	0.882
Calcium, ng/mL					0.832
Baseline	42 (100)	9.1 (0.8)	42 (100)	9.1 (0.8)	0.928
3 MPT	42 (100)	9.2 (0.7)	42 (100)	9.1 (0.7)	0.637
6 MPT	42 (100)	9.3 (0.7)	42 (100)	9.2 (0.7)	0.334
iPTH, pg/mL					0.226
Baseline	13 (31.0)	416.1 (260.0)	11 (26.2)	587.1 (302.3)	0.157^#^
3 MPT	12 (28.6)	753.7 (626.3)	13 (31.0)	542.6 (326.6)	0.312^#^
6 MPT	16 (38.1)	839.1 (633.4)	10 (23.8)	719.4 (540.5)	0.663
Phosphorus, mg/dL					0.334
Baseline	42 (100)	5.8 (1.3)	42 (100)	6.0 (1.3)	0.290
3 MPT	42 (100)	5.6 (1.2)	42 (100)	5.9 (1.4)	0.250
6 MPT	42 (100)	6.1 (1.6)	42 (100)	5.7 (1.4)	0.126

iPTH - intact parathyroid hormone; IV - intravenous; MPT - Months post-treatment or post-start of follow-up period, as applicable.

P values in bold represent the results of the General Linear Mixed Model (GLMM) for the overall analysis, while the remaining p values represent pairwise comparisons between the same timepoints of the study. No statistical differences were found between baseline and 3 MPT or 6 MPT.

* Median (Minimum - Maximum).

† No data were available for this parameter at baseline and 3 MPT.

# Not enough pairs available for comparison, which was made using non-paired t-test.

**Figure 1:**
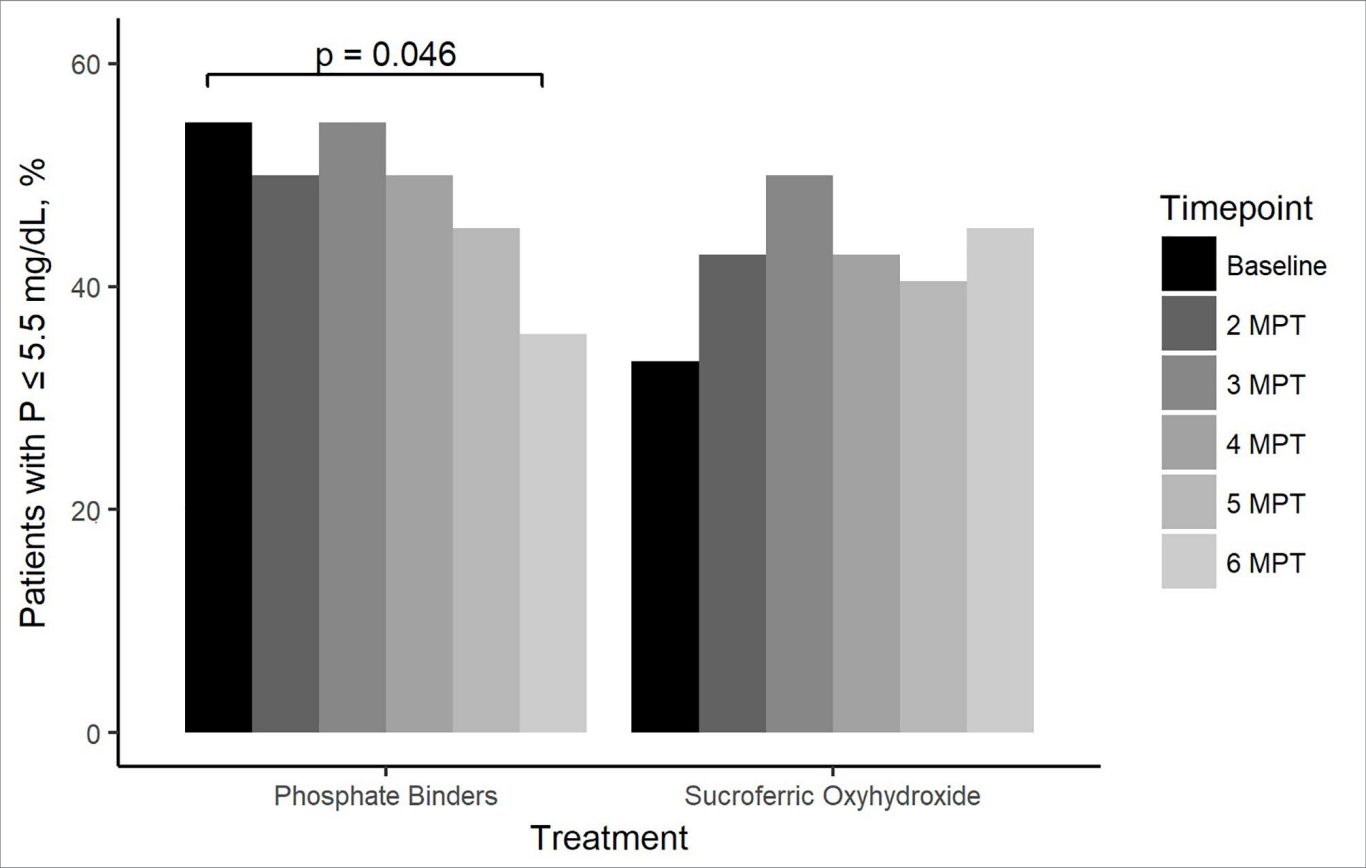
Percentage of patients that presented with phosphorus levels ≤ 5.5 mg/dL at each timepoint of the study, grouped by treatment. MPT: months post-treatment; P: phosphorus.

The proportion of patients with a serum phosphorus concentration ≤ 5.5 mg/dL decreased during the treatment with a PB (Figure 1). From baseline to six months post-treatment, the proportion of patients with serum phosphorus levels ≤ 5.5 mg/dL decreased from 54.8% to 35.7% (*p* = 0.046) (Figure 1). After switching to SO, the proportion of patients with serum phosphorus levels ≤ 5.5 mg/dL increased from 33.3% at baseline to 45.2% after six months of treatment (*p* = 0.225) (Figure 1), although the change was not statistically significant.

**Figure 2:**
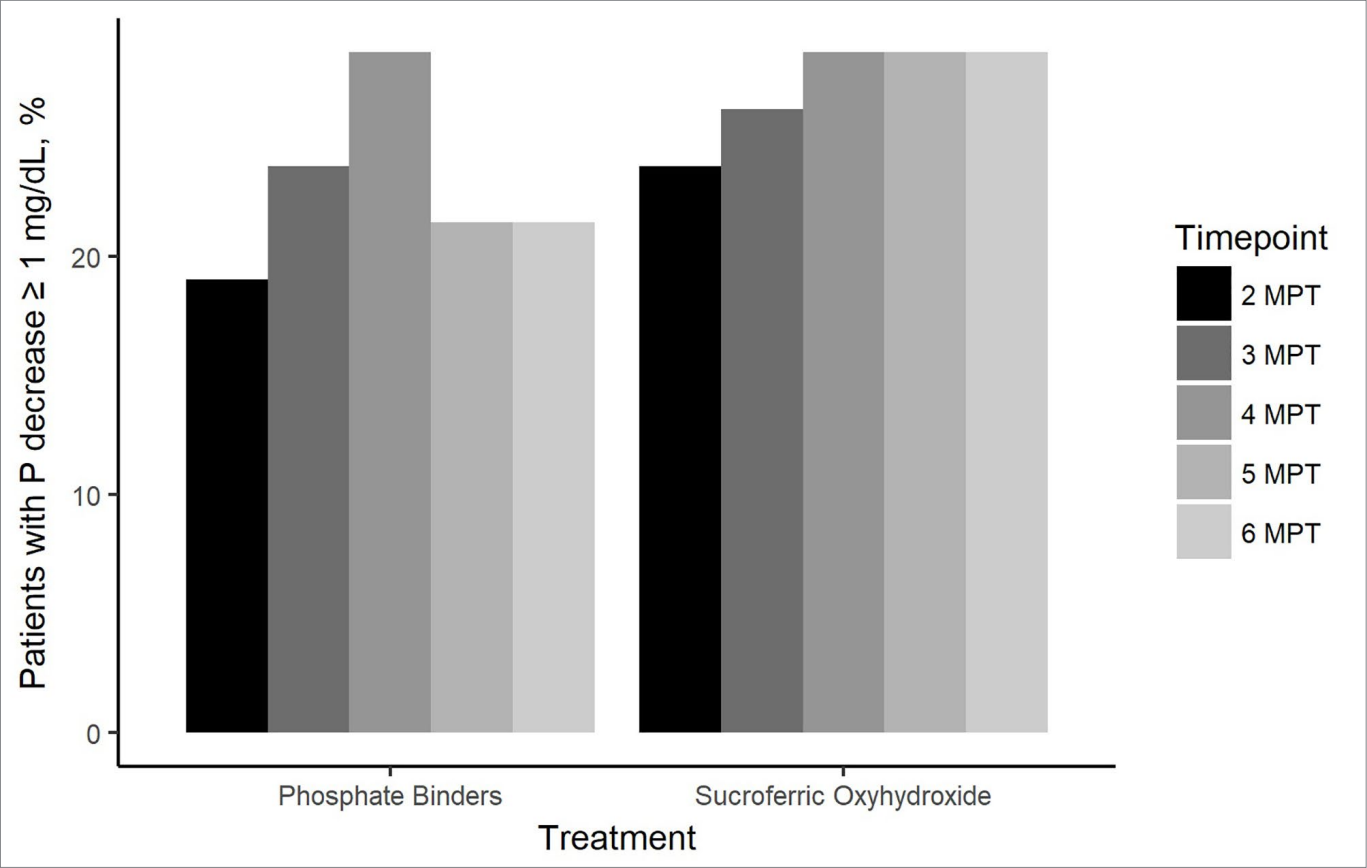
Percentage of patients with a decrease in phosphorus ≥ 1 mg/dL at each timepoint of the study as compared to baseline, grouped by treatment. MPT: months post-treatment; P: phosphorus.

During treatment with a PB, the percentage of patients with a reduction in serum phosphorus levels ≥ 1 mg/dL increased compared to baseline, from 19.0% at two months post-treatment to 21.4% at six months post-treatment (Figure 2). The proportion of patients with a decrease in serum phosphorus levels ≥ 1 mg/dL increased during the treatment with SO, from 23.8% at two months post-treatment to 28.6% at six months post-treatment (Figure 2).

**Figure 3:**
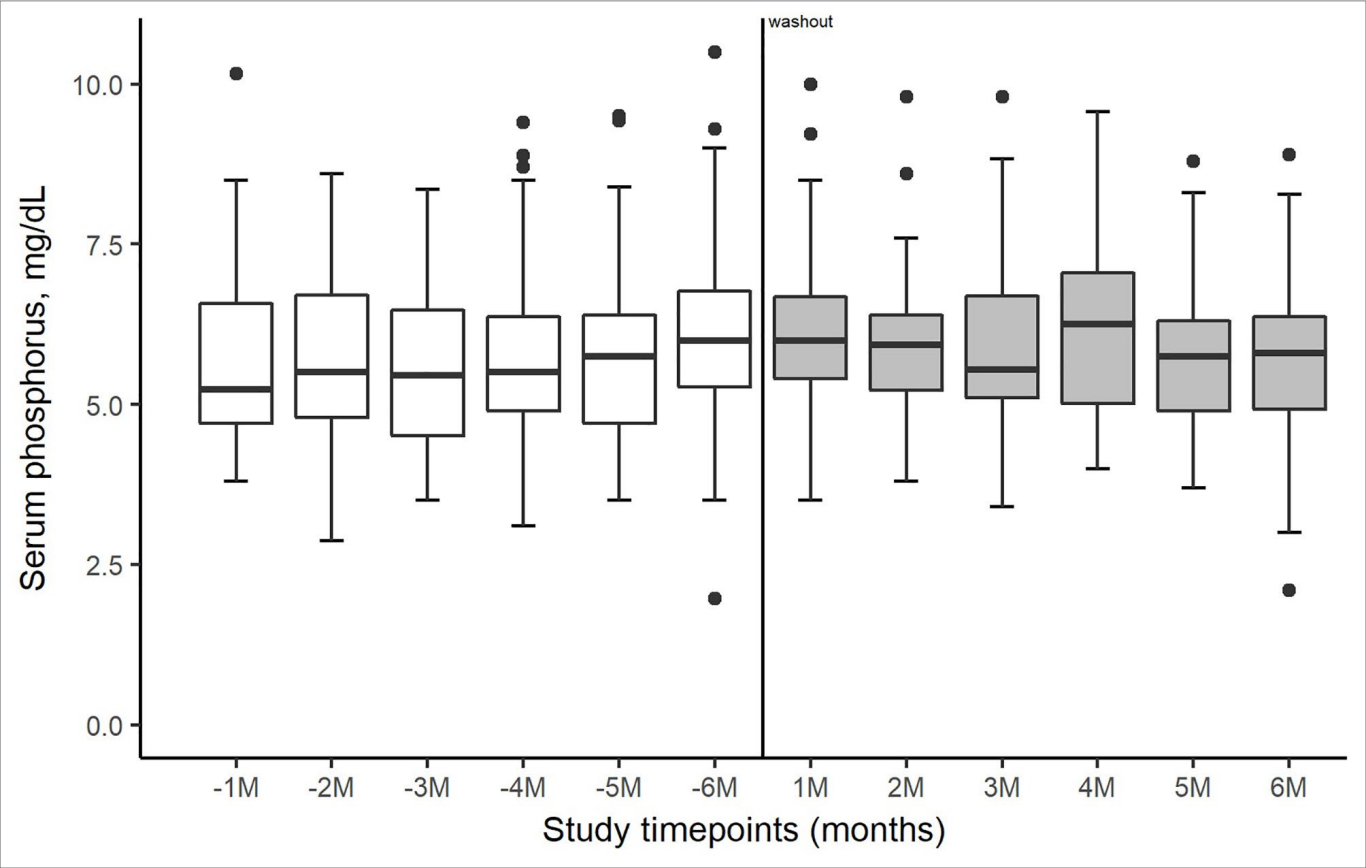
Side-by-side boxplots of phosphorus evolution over time from the start of the 6-month follow-up period of treatment with phosphate binders (-1M to -6M) until the end of the 6-month treatment period with sucroferric oxyhydroxide (1M to 6M).

Figure 3 depicts the overall change in serum phosphorus concentrations across the study period. Overall, SO controlled the phosphorus levels in the majority of the patients throughout the study period to the same extent as other PB (Figure 3).

## Discussion

The aim of this study was to assess the effectiveness of SO in controlling serum phosphorus levels in patients on online hemodiafiltration and determine the associated pill burden. This study showed that SO reduced the prescribed pill burden while effectively controlling serum phosphorus concentrations in patients on online hemodiafiltration.

Patients with CKD receiving dialysis are often required to take a large number of pills each day. A previous study reported that this patient group can have a median daily pill burden of 19 pills, with some patients taking more than 30 pills/day. PB accounted for up to 49% of the total daily pill burden.^7^ In the present study, it was observed that the median prescribed pills/day was reduced with 67% after switching from a PB to SO. At the same time, the serum phosphorus levels remained under control. These results are in line with what has been previously described in phase III studies, where Floege *et al*. reported that SO effectively controlled serum phosphorus levels in patients on dialysis, with a lower pill burden when compared to sevelamer carbonate.^10^^,^^11^^,^^14^ The high pill burden associated with CKD poses a challenge for the patients and contributes to non-adherence to treatment.^7^^,^^8^ Furthermore, decreased adherence to PB has been associated with higher serum phosphorus concentrations.^8^

One of the strengths of this study was that it included real-world data from difficult-to-treat patients, when at the time, no other treatment options were available. Six months after switching to SO, the percentage of patients with serum phosphorus levels ≤ 5.5 mg/dL increased 26% compared to baseline. At the same time, there was an increase in the proportion of patients with a reduction of serum phosphorus concentrations ≥ 1 mg/dL. It is possible that the lower pill burden led to higher adherence to treatment and better serum phosphorus control. In previous studies, adherence to treatment has been shown to be higher for SO compared to other PB such as sevelamer carbonate (82.6 vs 77.2%). Furthermore, non-compliance with treatment appeared to be more common in patients receiving sevelamer carbonate compared to SO (21.3 vs 15.1%).^11^ This study showed that treatment with SO controls serum phosphorus levels to the same extent as other PB but with a lower number of pills. The effect was observed after only 6 months of treatment with SO, which is promising and may potentially translate to better long-term compliance. However, this needs to be addressed in studies with a longer follow-up period.

A reduction in the dose and the number of patients receiving IV iron therapy after switching to SO was observed. SO is an iron-based PB and earlier studies have shown minimal uptake of iron by the gastrointestinal tract after administration of SO.^9^ The changes in iron parameters might have been due to iron uptake from SO, however this remains to be determined.

There are also limitations of this study derived from its retrospective design. Because data were obtained from patients’ medical records, we were not able to verify treatment adherence. In addition, clinical data were missing for some patients. In particular, clinical data on ferritin and iPTH levels were not available for several patients. In addition, the small number of patients may not be representative of the population of patients with CKD on online hemodiafiltration, making it difficult to ascertain the generalizability of the results. Nevertheless, this was a real-world study and we report successful phosphorus control following treatment with SO in this setting, which is consistent with results from phase II and phase III clinical studies.^10^^-^12

## Conclusions

In conclusion, SO controlled the serum levels of phosphorus in patients on hemodiafiltration to the same extent as other PB, with one third of the prescribed pill burden. A reduced pill burden may enhance therapeutic adherence and affect serum hyperphosphatemia in a positive manner and, hence, contribute to improved clinical outcome for patients with CKD on hemodiafiltration.
